# Spatial variability of excess mortality during prolonged dust events in a high-density city: a time-stratified spatial regression approach

**DOI:** 10.1186/s12942-017-0099-3

**Published:** 2017-07-24

**Authors:** Man Sing Wong, Hung Chak Ho, Lin Yang, Wenzhong Shi, Jinxin Yang, Ta-Chien Chan

**Affiliations:** 1 Department of Land Surveying and Geo-informatics, Hong Kong Polytechnic University, Kowloon, Hong Kong; 2 School of Nursing, Hong Kong Polytechnic University, Kowloon, Hong Kong; 3 Research Center for Humanity and Social Sciences, Academia Sinica, Taipei, Taiwan

**Keywords:** Spatial analytics, Extreme weather event, Dust mortality, Spatial variability, Community vulnerability, Geospatial modelling

## Abstract

**Background:**

Dust events have long been recognized to be associated with a higher mortality risk. However, no study has investigated how prolonged dust events affect the spatial variability of mortality across districts in a downwind city.

**Methods:**

In this study, we applied a spatial regression approach to estimate the district-level mortality during two extreme dust events in Hong Kong. We compared spatial and non-spatial models to evaluate the ability of each regression to estimate mortality. We also compared prolonged dust events with non-dust events to determine the influences of community factors on mortality across the city.

**Results:**

The density of a built environment (estimated by the sky view factor) had positive association with excess mortality in each district, while socioeconomic deprivation contributed by lower income and lower education induced higher mortality impact in each territory planning unit during a prolonged dust event. Based on the model comparison, spatial error modelling with the 1st order of queen contiguity consistently outperformed other models. The high-risk areas with higher increase in mortality were located in an urban high-density environment with higher socioeconomic deprivation.

**Conclusion:**

Our model design shows the ability to predict spatial variability of mortality risk during an extreme weather event that is not able to be estimated based on traditional time-series analysis or ecological studies. Our spatial protocol can be used for public health surveillance, sustainable planning and disaster preparation when relevant data are available.

## Background

Dust events are extreme pollution events that can induce adverse health effects in global cities, as studies have found for major cities in the United States, Australia, Asia and Europe [6, 12, 16, 31, 34, 40, 49]. Previous studies extensively applied temporally stratified models to quantify mortality risk during dust events, with promising results [31, 40, 49]; for example, one study showed that extreme events can lead to a 16% increase in dust mortality in a downwind city [31]. However, there has been no study to investigate the spatial variability of mortality risk during a prolonged dust event. In contrast to a lack of research on this issue, extensive environmental health studies on extreme weather and pollution have pointed out the necessity of predicting spatial variability of mortality and morbidity [2, 20, 23, 27, 29], for the purpose of measuring community vulnerability and public health planning. Estimating community vulnerability is particularly important to a high-density city, as the urban morphology of a high-density city influences air pollutant dispersion [58], resulting in extreme conditions with severe health risk, in particular at the district level. The prolonged effect of dust combined with urban morphology is expected to induce an additional effect on mortality in a downwind city.

In order to measure community vulnerability, both governmental health guidelines and previous studies have suggested investigating the environmental and socioeconomic factors that can additionally elevate the health risk [11, 17, 19, 24, 38, 41, 42]. Some studies have also proposed combining significant environmental and socioeconomic factors to pinpoint the hotspots of health risk [26, 44, 47, 50]. In order to enhance health planning to minimize adverse health effects of prolonged dust events, this study develops a set of protocols for (1) evaluating potential environmental and socioeconomic factors that can elevate mortality risk during a prolonged dust event, (2) including spatial influences of neighboring communities to adjust for environmental and socioeconomic effects on mortality risk, and (3) locating communities with higher mortality risk for disaster risk management during future dust episodes. The approach developed from this study could be applied to other regions where data on city-specific environmental and socioeconomic factors are available.

### Urban and climate settings of Hong Kong

Hong Kong is a typical high-density city located in a subtropical region. There have been ten reported days with dust events in the past decade in Hong Kong, including 2 days in 2006 (Apr 16–17, 2006), 4 days in 2009 (Apr 27–30, 2009), and 4 days in 2010 (Mar 23–26, 2010) [53, 54]. Two of these three dust events (8 of 10 days) were prolonged dust events with ≥3 consecutive dusty days. Significant mortality risk was observed on those days, with 7% increase in all-cause mortality and 7% increase in cardiorespiratory mortality during a dusty day. There was also significant air pollution during those dusty days, with average PM_10–2.5_ concentrations 147.6% higher than the days without dust. There is also an extreme population pattern in Hong Kong. The population density of Hong Kong is approximately 6500 persons per km^2^. The significant clustering of the urban population in Hong Kong potentially introduces significant intra-urban differences in mortality risk, due to the built environment and demographic structure [13, 33, 52].

## Methods

### Evaluation of community and environmental characteristics related to mortality risk

Mortality for dusty days and non-dusty days was calculated for each of 287 tertiary planning units (TPU), which is the smallest spatial unit in Hong Kong with mortality and census data available, in order to measure region-specific mortality risks across the entire territory of Hong Kong. The all-cause mortality dataset for dusty days was retrieved from mortality data of the Hong Kong Census and Statistics Department, based on 8 dust days (Apr 27–30, 2009 and Mar 23–26, 2010) associated with prolonged dust events (≥3 consecutive dust days), and by excluding all traffic-related deaths (ICD-10 codes V01–V99) during these prolonged dust episodes. Mortality on non-dusty days for each TPU was used to represent the baseline mortality. Deaths of the same weekday of four control weeks before and four control weeks after each dusty day were used to represent the mortality on non-dusty days, in order to minimize bias of seasonality and weekday/weekend effect; and this was divided by the numbers of control weeks for the purpose of comparison with the total mortality on all dusty days in Hong Kong.

To evaluate the potential community and environmental factors that influence mortality risk during a dust storm in Hong Kong, multivariate linear regression was firstly applied to estimate total mortality during the prolonged dust events. Six variables were included as independent variables to evaluate the environmental and socioeconomic effects on mortality in each TPU: (1) sky view factor (SVF), (2) percentage of vegetation, (3) land surface temperature (LST), (4) percentage of low education, (5) percentage of low income, and (6) percentage of elderly:

$$Total\;mortality\; = \;SVF\; + \;\% \;vegetation\; + \;average\;LST\; + \;\% \;low\;education\; + \;\% \;low\;income\; + \;\% \;elderly$$

where *total mortality* is the total mortality within 8 days of each TPU, *average SVF* is the average SVF of each TPU, % *vegetation* is the percentage of vegetation of each TPU, *average LST* is the average LST of each TPU, % *low education* is the percentage of low education population of each TPU, % *low income* is the percentage of low income population of each TPU and % *elderly* is the percentage of elderly of each TPU.

**Fig. 1:**
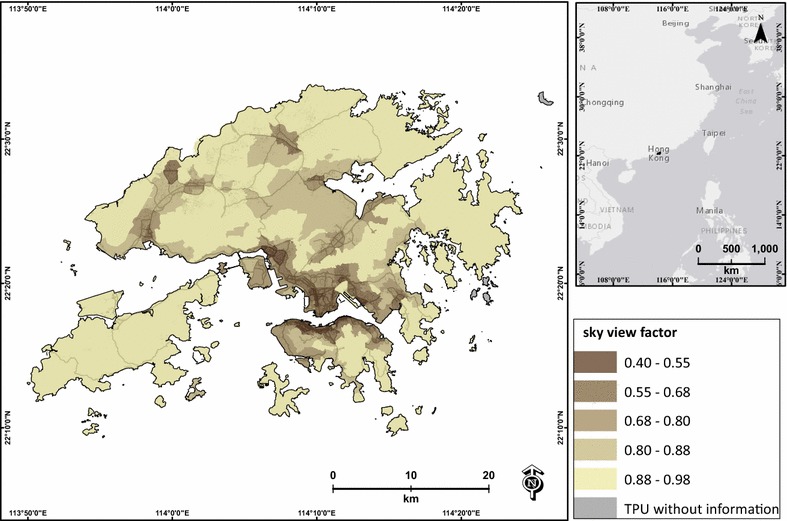
Average sky view factor of each TPU in Hong Kong

Urban geometric characteristics can be depicted by different parameterization indices such as building height, building density, frontal area index (FAI), planar area index (PAI), height/width ratio (H/W), and SVF. The SVF is an indicator representing combinations of building height, building density and topography [22]. The SVF is a ratio to measure the openness of a particular area within an urban setting and in general a terrestrial landscape, which has significant implications for the incoming and outgoing radiation [9]. SVF has been widely used in urban climate research [15, 21, 25, 28, 35, 45, 56], especially to improve spatial models of air pollution prediction [18, 39]. In this study, the SVF was used to locate high-density environments that can potentially trap air pollutants and prohibit air ventilation during prolonged dust events. Average SVF of each TPU was calculated based a raster-based SVF image of Yang et al. [56] derived from airborne Lidar data (Fig. 1). The SVFs at both rooftop and ground levels of this raster-based image were estimated and the spatial resolution of the airborne LIDAR data is 1 m. The building GIS data of Hong Kong were used to calculate the SVF for vertical facets using the planar area index (PAI) and the frontal area index (FAI).

**Fig. 2:**
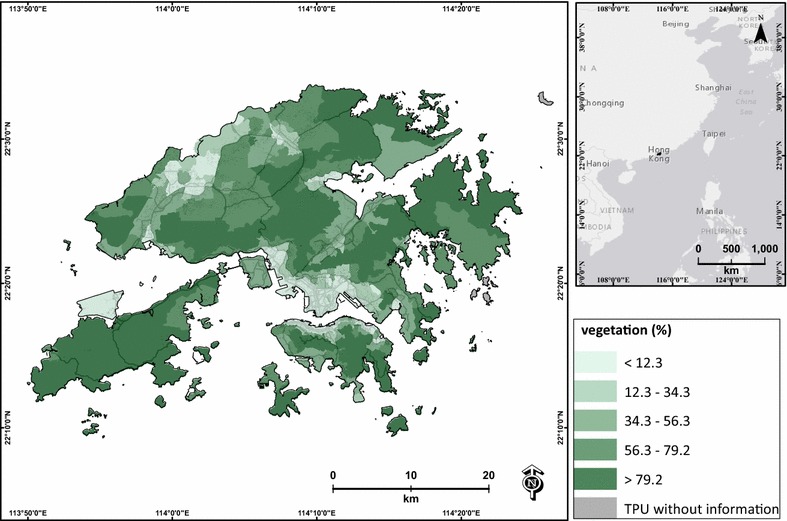
Average vegetation cover of each TPU in Hong Kong

Vegetation coverage (measured in percentage) can potentially influence or absorb ground-level air pollution in each TPU. In this study, the territory-wide vegetation coverage was estimated using the land use and land cover map derived from the Planning Department of Hong Kong (Fig. 2).

**Fig. 3:**
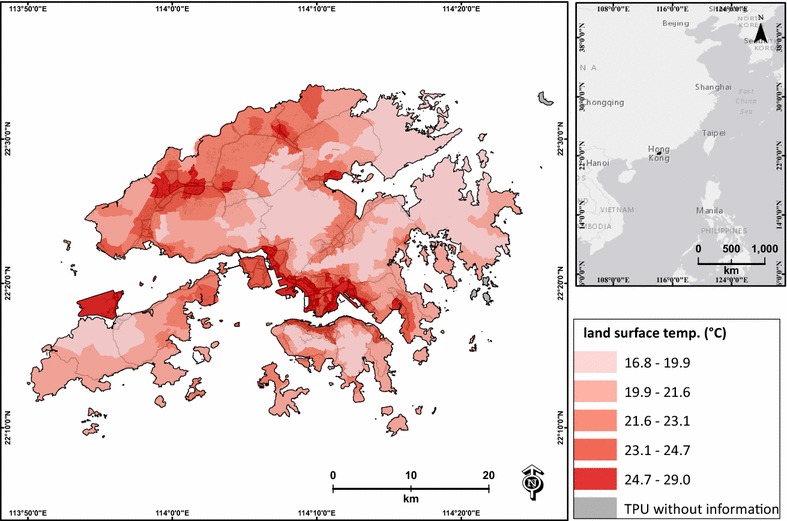
Average land surface temperature of each TPU in Hong Kong

Land surface temperature (LST) images are commonly used to represent spatial variations of surface temperature that can affect health risk [29, 36, 55]. Landsat Thematic Mapper TM 5 on March 25, 2010 was used to estimate LST to demonstrate typical temperature variations during prolonged dust events in Hong Kong. Average LST (Fig. 3) was estimated using an improved urban emissivity model based on the SVF [57].

**Fig. 4:**
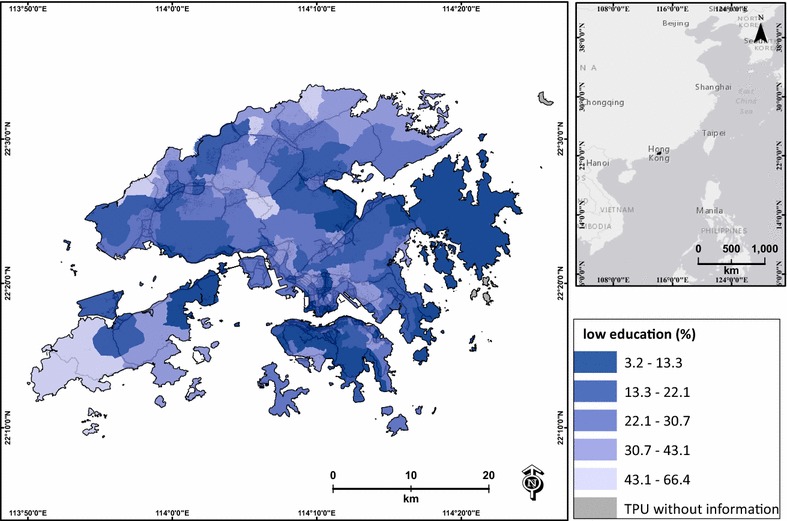
Percentage of low-education population (primary school graduate or below) of each TPU in Hong Kong

**Fig. 5:**
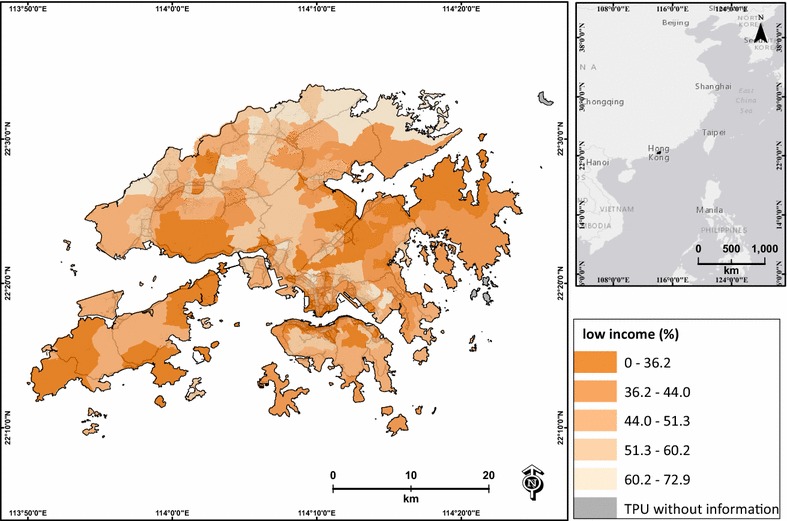
Percentage of low-income population (monthly income lower than HKD $10,000) of each TPU in Hong Kong

**Fig. 6:**
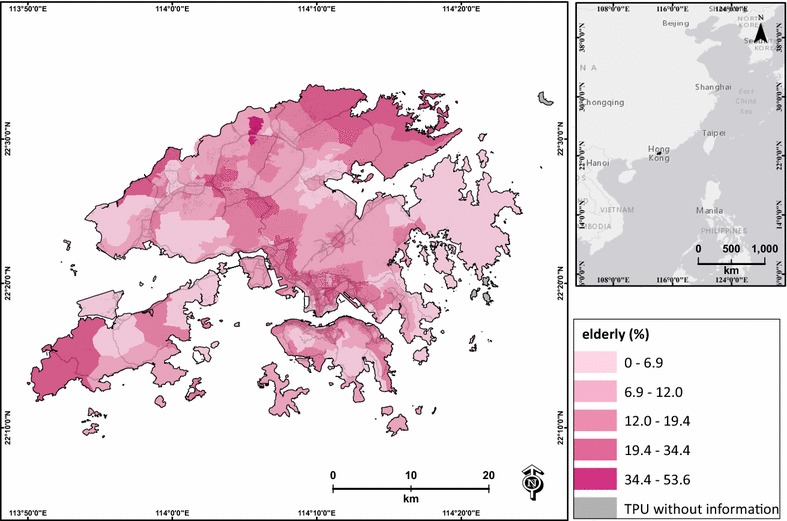
Percentage of elderly (age ≥65) of each TPU in Hong Kong

Lower education is associated with higher social vulnerability during air pollution events [32, 52], and lower income is related to low socioeconomic status, which may induce adverse health effects on a day with heavy pollution [52]. The elderly are identified as one of the major age groups that are highly vulnerable during days with heavy air pollution [8, 32]. Therefore, the percentages of low education, low income and elderly were extracted from the 2006 census data of Hong Kong, and were used to represent the socioeconomic influence of each TPU. The percentage of low education was calculated based on the percentage of persons who had a primary school education or less (Fig. 4). The percentage of low income was the percentage of persons who were unpaid or had monthly income lower than HKD$10,000 (Fig. 5). The percentage of elderly was the percentage of persons aged ≥65 in each TPU (Fig. 6). Two TPUs with missing data of low education, low income and elderly were excluded from this study.

**Fig. 7:**
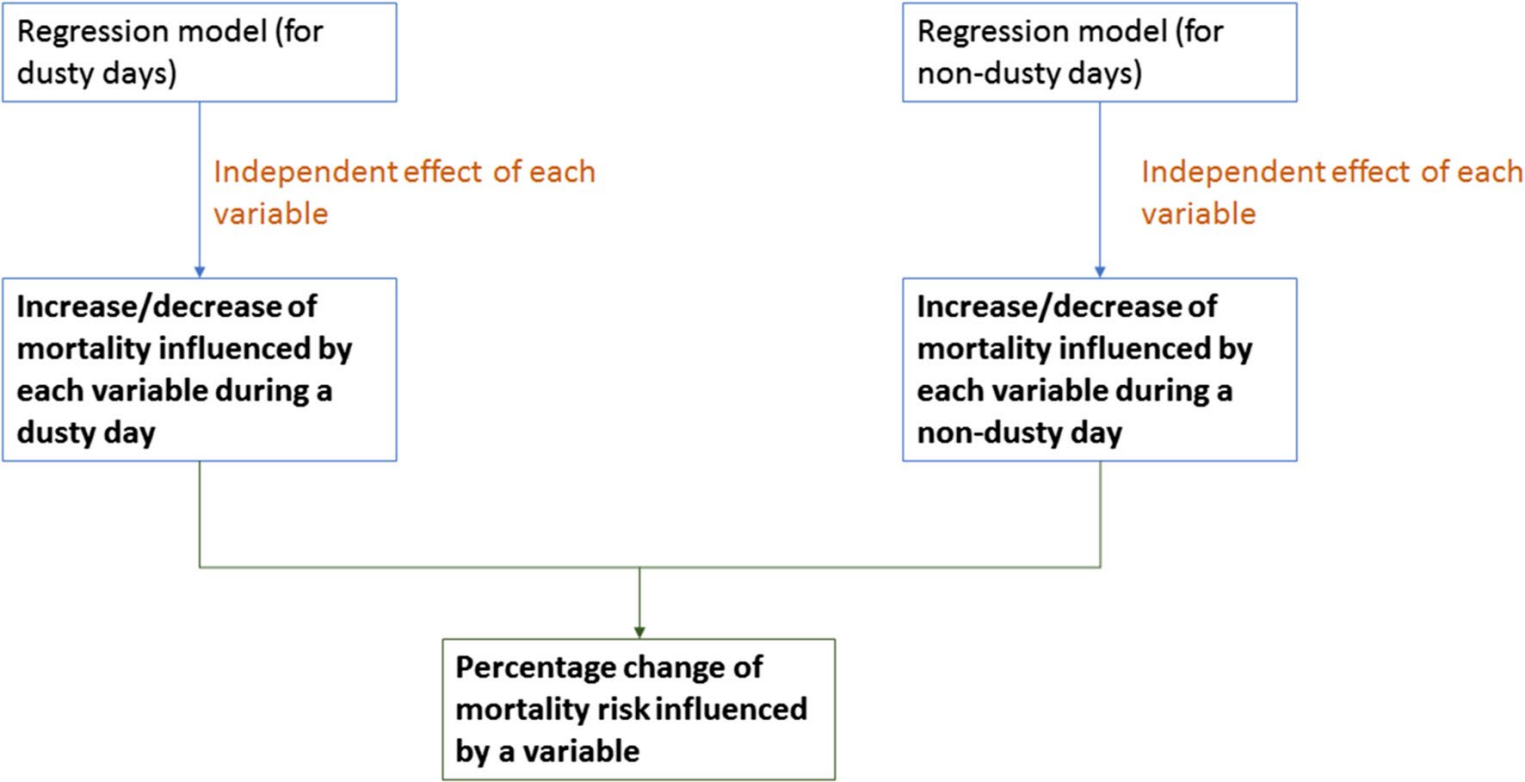
Flow diagram of the model development and risk estimation

Finally, the predicted total mortality and the 95% confidence interval (CI) were estimated to represent the additional effect of mortality risk on both dusty days and non-dusty days (baseline) from the spatial variability of each variable. Excess mortality between dusty days and non-dusty days contributed by each spatial factor was also reported in this study (Fig. 7).

### Including neighboring effects for mortality risk estimation

To include neighboring effects on mortality risk of each TPU, a spatial error model was applied and was compared with the results of multivariate linear regression. The spatial error model incorporated spatial autocorrelation in a regression error term (Lambda) to adjust spatial dependence in a multivariate linear regression model [3]. In our study, the spatial error model weights the neighboring TPUs to spatially adjust socioeconomic and environmental influences on total mortality. To spatially weight the TPUs, we applied the queen contiguity method. This method weights all spatial neighbors with shared borders and corners [4], based on a spatial distance with the order of contiguity. This study applied the queen contiguity method to adjust the mortality risk of each TPU based on all surrounding TPUs. To evaluate the appropriate spatial distance for adjustment, the 1st to 3rd orders of contiguity (lag 1–lag 3) were used to estimate total mortality and for comparison with the linear results. All significant environmental and socioeconomic variables were used to construct the spatial error models with the 1st–3rd order of contiguity, and the multivariate linear regression for comparison. The Akaike information criterion (AIC) [10] was then adopted to compare the models, in which lower AIC indicated better model performance. Total mortality and CIs representing the additional effects of mortality risk from the spatial variability of each variable were also reported, and the differences between models were further evaluated. Finally, predicted mortality change (increase or decrease) in numbers of deaths of each TPU from the appropriate models for both dusty and non-dusty days were used to illustrate the spatial variability of relative mortality across Hong Kong during prolonged dust events.

## Results

### Contributions of socioeconomic and environmental influences to mortality risk

There were in total of 802 decedents reported on all case days, and 6331 decedents reported on all control days. Based on the multivariate linear models, the SVF, percentage of low education, percentage of low income, and percentage of elderly show varied contributions to local mortality risk of each TPU during prolonged dust events (Table 1). Among all, percentage of low income is the highest risk factor of a community during prolonged dust events. A TPU with 10% more low-income population is found to have 12.5% higher mortality during a dusty day than a non-dusty day. At a TPU with 10% more population who education level was primary school or below, there is also 6.3% higher mortality during prolonged dust events than days without dust. In contrast, SVF has a negative association with mortality risk. A TPU with 10% higher SVF has 5.3% less mortality during a day with a dust storm. This indicates that a TPU with a high-density built environment generally has higher risk during prolonged dust events, while lower-density environments with higher SVF have less mortality risk.

**Table 1:** Influences of community factors on excess mortality

Variables	Predicted total mortality		
	Change in number of deaths on days with prolonged dust events (95% confidence intervals)	Baseline: number of deaths on days without prolonged dust events (95% confidence intervals)	Excess mortality (%)
SVF (in 10%)	−2.0 [−2.6, −1.3]*	−1.9 [−2.5, −1.3]*	−5.3
% vegetation (in 10%)	0.0 [−0.4, 0.4]	0.0 [−0.3, 0.4]	0
LST (in 1 °C)	−0.1 [−0.6, 0.4]	−0.1 [−0.5, 0.3]	0
% low education (in 10%)	1.7 [0.8, 2.5]*	1.6 [0.9, 2.3]*	6.3
% low income (in 10%)	0.9 [0.2, 1.6]*	0.8 [0.2, 1.4]*	12.5
% elderly (in 10%)	−2.4 [−3.6, −1.1]*	−2.2 [−3.2, −1.1]*	−9.1

* Are the results with significant *p* values (<0.05)

In addition, there is no observation of an increase of total mortality in the TPUs with a higher percentage of elderly. Similar results have been found in other Hong Kong studies; for example, Chan et al. [13] estimated community vulnerability with census data and found that those aged ≥75 had significantly lower mortality risk than those aged <75, especially for the male population. Spatial differences in temperature and vegetation did not have significant contributions to mortality risk during prolonged dust events.

### Including spatial influences for mortality risk estimation

Compared to the non-spatial model for dusty days using all variables with an AIC of 1692.3, the non-spatial model using only significant environmental and socioeconomic variables (SVF, % lower education, % lower income and % elderly) has a lower AIC of 1689.6, indicating a better model for prediction. By using all significant environmental and socioeconomic variables, it is observed that inclusion of neighboring effects as spatial influential factors has enhanced mortality risk estimation. By comparing all models for predicting mortality during dusty days with and without incorporating spatial autocorrelation (Table 2), the model considering the 1st order of queen contiguity (lag 1) has the best performance. The AIC of this lag-1 model is 1679.97, and it is the lowest among the others. There is a positive value of the regression error term of the lag-1 model (Lambda: 0.3), indicating that including neighboring effects of surrounding TPUs results in less spatial error for mortality risk prediction. It is also important to note that only including the 1st- or 2nd-order queen contiguity in modelling can enhance mortality risk estimation for prolonged dust events in Hong Kong. The 3rd-order queen contiguity does not improve the modelling, based on Lambda reported with the spatial error model. We also repeated the analyses for mortality predictions of non-dusty days (Table 3). We found similar results for model comparison, with the lag-1 model the best for predicting mortality during days without prolonged dust events (AIC: 1582.04).

**Table 2:** Comparison of spatial and non-spatial models for predicting total mortality during days with prolonged dust events

Variables	Multivariate linear: predicted mortality on dusty days (95% confidence intervals)	Spatial error (lag 1): predicted mortality on dusty days (95% confidence intervals)	Spatial error (lag 2): predicted mortality on dusty days (95% confidence intervals)	Spatial error (lag 3): predicted mortality on dusty days (95% confidence intervals)
SVF (in 10%)	−1.8 [−2.2, −1.4]*	−2.0 [−2.5, −1.5]*	−2.0 [−2.5, −1.6]*	−1.8 [−2.2, −1.4]*
% low education (in 10%)	1.6 [0.8, 2.4]*	1.6 [0.7, 2.5]*	1.5 [0.7, 2.4]*	1.5 [0.7, 2.4]*
% low income (in 10%)	0.9 [0.2, 1.6]*	0.7 [0.0, 1.4]	0.8 [0.1, 1.5]*	0.9 [0.2, 1.6]*
% elderly (in 10%)	−2.3 [−3.5, −1.1]*	−2.1 [−0.8, −3.4]*	−2.1 [−3.3, −0.9]*	−2.2 [−3.4, −1.0]*
Lambda	N/A	0.3 [0.1, 0.4]*	0.4 [0.1, 0.6]*	0.1 [−0.2, 0.4]
AIC	1689.6	1679.97	1682.95	1689.03

* Are the results with significant *p* values (<0.05)

**Table 3:** Comparison of spatial and non-spatial models for predicting total mortality during days without prolonged dust events

Variables	Multivariate linear: predicted mortality on non-dusty days (95% confidence intervals)	Spatial error (lag 1): predicted mortality on non-dusty days (95% confidence intervals)	Spatial error (lag 2): predicted mortality on non-dusty days (95% confidence intervals)	Spatial error (lag 3): predicted mortality on non-dusty days (95% confidence intervals)
SVF (in 10%)	−1.7 [−2.1, −1.4]*	−1.9 [−2.3, −1.5]*	−2.0 [−2.4, −1.6]*	−1.8 [−2.1, −1.4]*
% low education (in 10%)	1.5 [0.8, 2.2]*	1.5 [0.7, 2.2]*	1.4 [0.7, 2.2]*	1.4 [0.7, 2.1]*
% low income (in 10%)	0.8 [0.2, 1.4]*	0.6 [0.0, 1.2]	0.7 [0.1, 0.3]*	0.8 [0.2, 1.4]*
% elderly (in 10%)	−2.1 [−3.1, −1.1]*	−1.8 [−2.9, −0.7]*	−1.9 [−2.9, −0.9]*	−1.9 [−3.0, −0.9]*
Lambda	N/A	0.3 [0.2, 0.5]*	0.4 [0.2, 0.6]*	0.2 [−0.1, 0.5]
AIC	1612.48	1582.04	1585.25	1610.28

* Are the results with significant *p* values (<0.05)

By using the lag-1 model to include the neighboring effects (Table 4), areas with 10% higher SVF will have 5.3% less mortality risk than TPUs during a prolonged dust event, while a TPU with 10% more low-education population will have 6.7% higher mortality during prolonged dust events compared to non-dusty days, with all these reaching statistical significance.

**Table 4:** Influences on excess mortality based on the best spatial regression models

Variables	Spatial error (lag 1): predicted total mortality on dusty day (95% confidence intervals)	Spatial error (lag 1): predicted total mortality on non-dusty day (95% confidence intervals)	Excess mortality (%)
SVF (in 10%)	−2.0 [−2.5, −1.5]*	−1.9 [−2.3, −1.5]*	−5.3
% low education (in 10%)	1.6 [0.7, 2.5]*	1.5 [0.7, 2.2]*	6.7
% low income (in 10%)	0.7 [0.0, 1.4]	0.6 [0.0, 1.2]	16.7
% elderly (in 10%)	−2.1 [−3.4, −0.8]*	−1.8 [−2.9, −0.7]*	−16.7

* Are the results with significant *p* values (<0.05)

**Fig. 8:**
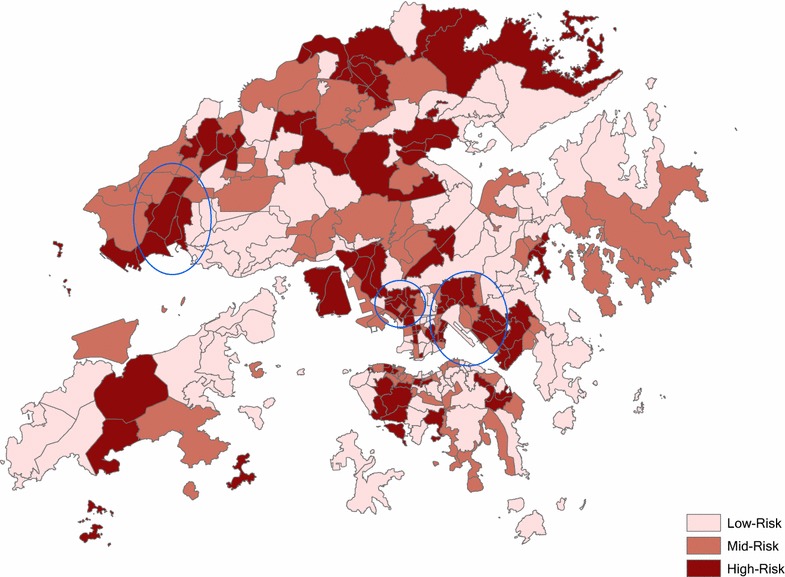
Mortality risk of each TPU during prolonged dust events in Hong Kong. *Blue circles* are the areas with a high-density environment and high socioeconomic deprivation (Tuen Mun, Sham Shui Po, Wong Tai Sin and Kwun Tong)

Based on the comparison of spatial and non-spatial models, we applied a spatial error model incorporating the 1st order of queen contiguity to predict total mortality on dusty days and non-dusty days, and a predicted change of total mortality as relative risk of each TPU is reported in this study (Fig. 8). The mortality risk map indicates that rural areas with low-density environments have a potential decrease in mortality during prolonged dust events compared to non-dusty days. In contrast, the TPUs with high-density environments and high socioeconomic deprivation, such as TPUs in Tuen Mun, Sham Shui Po, Wong Tai Sin and Kwun Tong, generally have a higher increase in mortality during prolonged dust events compared to non-dusty days. These TPUs are predicted to have 0.1–0.5 more deaths in a period of 8 dust days than the control periods, controlling for SVF, percentage of low education, percentage of low income, percentage of elderly, and spatial autocorrelation.

## Discussion

This study applied a spatial regression approach to estimate spatial variability of mortality risk across a high-density city during prolonged dust events. Based on this approach, the influence of the built environment is highlighted by the negative association between SVF and mortality increase. This result indicates that high-density urban areas may trap air pollutants during days with dust storms, resulting in poorer air quality and severely increasing health risk; while areas with more openness allow better air ventilation and dispersion, therefore less health risk attributable to air pollution can be found in these areas during dusty days. Influence from socioeconomic deprivation is also determined by the positive association between total mortality, percentage of low education, and percentage of low income. In contrast, percentage of elderly of a TPU does not have a positive association with mortality risk. This might be due to the presence of more health facilities in communities with higher percentages of elderly, which reduces the mortality risk of such neighborhoods, while elderly in TPUs with lower percentages of older population may not benefit from such facilities, therefore increasing their risk. In conclusion, these findings are innovative, because previous studies only temporally stratified the dust mortality [12, 14, 31, 40, 49], without understanding the intra-urban difference in mortality risk during dust events.

In the context of spatial health planning, health risk mapping can characterize vulnerability of specific populations in a specific region [24], for the purpose of supporting health authorities, policymakers, and city officials to determine future health protocols in different communities [1]. This mapping technique has been widely used along with governmental actions to develop public health surveillance. For example, the City of Toronto in Canada initiated a heat vulnerability mapping project for minimizing summer risks [44], and Vancouver Coastal and Fraser Health Authorities gave impetus to the development of the Vancouver Area Neighborhood Deprivation Index (VANDIX) for general health risk estimation [7]. Mapping mortality risk adjusted for environmental and socioeconomic factors can help target a single disaster episode for comprehensive health planning. This is necessary because the general health vulnerability index can be somewhat useful, but may not be able to fully describe the spatial variability of a particular health risk [43]. One example is that VANDIX is related to heat mortality in the Vancouver area, but it is necessary to adjust it to pinpoint heat risks with accuracy [27]. Therefore, previous health studies indicate the need to calibrate spatial vulnerability assessments with health outcome data [5, 11, 26, 47, 50], while mortality data will be the most appropriate dataset for demonstrating disaster episodes. Therefore, mapping mortality risk during prolonged dust events is essential, since such spatial assessment can be used for local government action, as well as serving as a regional protocol for developing similar health indices in other cities.

Furthermore, previous research mostly developed health indices based on a simple spatial overlay technique [50]; there were issues related to spatial autocorrelation that these studies did not consider, and that might create potential biases in results. Our study adopting spatial error modelling is test-proven, with promising results showing that spatial autocorrelation can significantly improve the accuracy of predicting mortality during dust episodes. Its findings are similar to those of other spatial epidemiologic literature showing that analysis with spatial autocorrelation can help predict spatial variability of health risks [37, 51].

One limitation of this study is that the prolonged dust events were isolated episodes in Hong Kong. Based on only two dust events in Hong Kong, it was not able to employ time-series analysis for a more comprehensive spatio-temporal assessment. Application of an alternative method such as a time-stratified approach for estimating the standard mortality ratio of each small neighborhood is also problematic, since comparing rare death cases on non-dusty days in each neighborhood may create extreme estimation, resulting in statistical bias. To avoid the statistical bias, previous health studies generally applied spatial delineation techniques to stratify socioeconomic or environmental data by groups [27, 48, 52]. This method can capture spatial differences between groups, but is still insufficient to estimate the individual risk of each district. Our approach is applicable for the present case study, because we applied spatial regression to predict mortality on both dusty and non-dusty days for comparison. With the support of spatial regressions to compare total mortality between dusty and non-dusty days, the results of this study can be used to demonstrate the additional mortality effect in each district due to spatial variability of environmental and socioeconomic factors during the isolated but fatal dust events.

For future study, inclusion of spatial data on air pollution exposure may enhance mortality risk mapping. However, existing pollution mapping methods such as land use regression are limited by the spatiotemporal coverage of the data, which may not be able to demonstrate extreme cases such as a prolonged dust event. In addition, there is an accuracy issue in using such mapping methods in a high-density city, because a complex urban built environment influences air ventilation, and as a result produces bias in pollution mapping [46]. Misuse of air pollution maps for a spatial study can induce a significant ecological fallacy, especially since community vulnerability is already influenced by the adverse effect due to aggregate-level data, instead of the association with individual-level response [13]. In order to tackle this issue, some studies have started to use moderate-resolution satellite images for mapping Aerosol Optical Depth (AOD) or Aerosol Optical Thickness (AOT) in order to demonstrate spatiotemporal variation in air pollution. However, there are varied associations between AOD/AOT and particulate matters (the main components of dust), depending on the size of particulate matters and spatial locations. While fine particulate matter is a common pollutant contributing to health risk in typical non-dust scenarios [30], there are also studies finding that PM_10_ or PM_10–2.5_ concentration may severely increase the mortality risk during a dusty day [16, 31, 40]. Therefore, further investigation is needed on how to use AOD/AOT to map spatiotemporal variations of both fine and coarse particulate matters for determining how air pollution exposures actually influence mortality risk during a dusty day. A future study should, therefore, combine an existing city-based mapping method with satellite images to improve the spatio-temporal modelling of air pollution exposure, at the same time increasing the spatial quality, because moderate or low spatial resolution of satellite images itself may increase potential spatial biases of modelling.

## Conclusion

This study applied a spatial regression approach to estimate the spatial variability of mortality during prolonged dust events. The results indicated that spatial difference in built environment (SVF) and socioeconomic status (low education and low income) will increase the mortality in a community during dust events. This study also demonstrates there is a need to include spatial autocorrelation in modelling in order to improve the accuracy of prediction. Finally, the mortality risk map can be used to locate at-risk communities and vulnerable populations for developing health protocols in Hong Kong.

## Abbreviations

FAI: frontal area index (FAI)
PAI: planar area index (PAI)
H/W: height/width ratio
LST: land surface temperature
CI: confidence interval
AIC: Akaike information criterion
SVF: sky view factor
TPU: tertiary planning unit
VANDIX: Vancouver Area Neighborhood Deprivation Index
AOD: aerosol optical depth
AOT: aerosol optical thickness
PM: particulate matter
